# Association between appropriateness of coronary revascularization and quality of life in patients with stable ischemic heart disease

**DOI:** 10.1186/1471-2261-14-137

**Published:** 2014-10-04

**Authors:** Harindra C Wijeysundera, Feng Qiu, Paul Fefer, Maria C Bennell, Peter C Austin, Dennis T Ko

**Affiliations:** Schulich Heart Centre, Division of Cardiology, Sunnybrook Health Sciences Centre, University of Toronto, Toronto, Ontario Canada; Institute of Health Policy, Management and Evaluation, University of Toronto, Toronto, Ontario Canada; Institute for Clinical Evaluative Sciences (ICES), Toronto, Ontario Canada; Li Ka Shing Knowledge Institute of St. Michael’s Hospital, Toronto, Ontario Canada; Leviev Heart Center, Sheba Medical Center, Tel Hashomer and Tel Aviv University, Ramat Aviv, Israel

**Keywords:** Quality of life, Angina, Appropriateness

## Abstract

**Background:**

The relationship between appropriateness score, treatment strategy and quality of life (QOL) among patients with stable ischemic heart disease (SIHD) is not known. In this prospective cohort study, we evaluated changes in generic and cardiac-specific quality of life in patients with documented SIHD, comparing patients with revascularization versus those with medical therapy alone, stratified by their appropriateness scores.

**Methods:**

Consecutive patients with SIHD undergoing elective coronary angiogram from November 1^st^ 2008 to December 1^st^ 2009 completed the Seattle Angina Questionnaire (SAQ) and EQ-5D at the time of procedure and at 1 year. The appropriateness for coronary revascularization was determined at the time of coronary angiography.

**Results:**

Our final cohort consisted of 425 patients, 69.4% of whom underwent revascularization. In the overall cohort, 272 (64.0%) had appropriate indications for revascularization, while 57 (13.4%) had inappropriate indications and 96 (22.6%) had uncertain indications. On average, patients improved in most QOL domains, regardless of treatment strategy and appropriateness score. In patients with appropriate indications, revascularized patients had greater improvements in both generic (0.073; 95% CI 0.003-0.144; p-value 0.04) and disease-specific indices, including angina stability (14.6; 95% CI 0.85-28.3; p-value 0.04), physical limitation (9.3; 95% CI 0.71-17.8; p-value 0.03) and disease perception (12.7; 95% CI4.3-21.1; p-value 0.003) compared to medically treated patients. However, patients with uncertain and inappropriate indications also had improvements in physical limitation and disease perception with revascularization compared to medical therapy.

**Conclusions:**

Patients who had appropriate revascularization derived the greatest improvement in QOL compared with medical therapy.

**Electronic supplementary material:**

The online version of this article (doi:10.1186/1471-2261-14-137) contains supplementary material, which is available to authorized users.

## Background

Landmark randomized controlled trials have consistently shown no difference in death or myocardial infarction between patients with stable ischemic heart disease (SIHD) who are treated with optimal medications alone compared to those treated with revascularization, with either coronary artery bypass grafting (CABG) or percutaneous coronary intervention (PCI) [1–3]. Despite this, there is wide regional variation in the use of coronary revascularization, suggesting the possible misuse of these invasive therapies [4, 5].

The appropriate use criteria for coronary revascularization were designed to assist physicians balance the potential benefits of revascularization against its potential harms [6, 7]. Organized jointly by six international cardiovascular societies, an expert panel used a modified Delphi process to categorize clinical scenarios into appropriate, uncertain and inappropriate indications for coronary revascularization [6, 7]. The rationale for the appropriate use criteria in identifying candidates for revascularization will be strengthened if one demonstrates a strong relationship between improved outcomes and revascularization decisions based on the appropriateness score. Arguably, the most important indication for revascularization in SIHD is reducing symptomatic angina and improving quality of life, given the equivalence between optimal medical therapy and revascularization in reducing hard clinical outcomes. To the best of our knowledge, there are no data on the relationship between the appropriateness of revascularization and its impact on quality of life.

Accordingly, our objective in this prospective cohort study was to address this gap in knowledge by evaluating changes in generic and cardiac-specific quality of life over one year in patients with documented SIHD based on non-emergent coronary angiography, comparing patients with revascularization versus those with medical therapy, stratified by their appropriateness scores.

## Methods

This study was approved by the Institutional Research Ethics Boards at Sunnybrook Health Sciences Center at the University of Toronto. All enrolled patients provided informed consent to participate in the study.

### i. Data source

Our study cohort consisted of consecutive patients with stable chronic coronary artery disease who underwent coronary angiography at the Schulich Heart Center, Sunnybrook Health Sciences Centre in Toronto, Ontario, Canada. Patients were recruited from November 1^st^ 2008 to December 1^st^ 2009. We excluded patients whose indications for angiography were myocardial infarction, acute coronary syndrome, or valvular heart disease. We also excluded patients under the age of 20 years and those with normal coronary arteries, or only mild coronary artery disease, defined as stenosis less than 70% in severity (or less than 50% in the left main artery).

Socio-demographic features, co-morbidities, baseline medications, coronary anatomy, left ventricular (LV) function, as well as pre-procedural stress testing were collected. Patients completed two self-administered quality of life surveys, the Seattle Angina Questionnaire (SAQ) and the EQ-5D just prior to the baseline angiogram, with follow-up at 1 year. The questionnaires were mailed to patients for completion, with telephone reminders provided. Abstracted data were linked using unique patient identifiers to population-based administrative databases housed at the Institute for Clinical Evaluative Sciences (ICES). Death was ascertained through linkage with the Ontario Registered Persons Database (RPDB). Subsequent cardiac procedures were determined through linkage with the Canadian Institute for Health Information Discharge Abstract Database (CIHI-DAD) and Same-Day Surgery database (CIHI-SDS). These contain data on all in-hospital and outpatient procedures, as well as acute hospitalizations.

### ii. Measures

#### a. Seattle Angina Questionnaire (SAQ)

The SAQ is a 19-item descriptive, self-administered questionnaire that focuses on impairments in health unique to coronary disease, across five dimensions: physical limitation, anginal stability, anginal frequency, disease perception and treatment satisfaction [8]. Each dimension is scored and analyzed separately on a continuous scale with a range from 0 to 100, with higher scores indicating better quality of life [8]. A clinically relevant improvement in the SAQ that reflects a change that is perceptible to patients is an improvement of 10 points, while a substantial improvement in clinical status is considered to be an increase of 20 points [8].

#### b. EQ-5D

The EQ-5D measures the preference for health status using a single index score that has two anchors, with a low score of 0 representing death, and an upper ceiling of 1, representing perfect health [9]. The EQ-5D covers five dimensions of health: mobility, self-care, usual activities, pain/discomfort, and anxiety/depression [9]. Each dimension is measured using a three-level ordinal scale and a unique health state is defined by a combination of values on each of the five dimensions [9]. Each of the 243 potential health states is assigned a score by applying a country-specific tariff. US tariffs were used in this study [9]. To provide clinical context, previous studies have found that Canadian Cardiovascular Class (CCS) 2 is associated with an EQ-5D score of 0.77, while CCS 4 is associated with a score of 0.72 [10].

### iii. Treatment category

Patients were assigned to the revascularization category if they underwent a PCI or CABG within 90 days of index angiogram. All other patients were assigned to the medical therapy category.

### iv. Appropriateness category

The appropriateness for coronary revascularization was determined based on data collected at the time of the index angiogram. For this study, we used the 2009 appropriate use criteria, and restricted our analyses to the 59 indications related to SIHD [7]. Briefly, each patient was assigned an appropriateness score from 1 to 9, based broadly on coronary anatomy (single versus double versus triple vessel coronary artery disease), the severity of symptoms (based on Canadian Cardiovascular (CCS) class), the intensity of anti-anginal medications and the extent of ischemia on non-invasive stress testing [6, 7]. For each patient, the score was calculated automatically using a computer algorithm. Patients were categorized into those with appropriate indications for revascularization (score of 7–9), uncertain indications (score of 4–6) and inappropriate indications (score of 1–3). Patients that could not be assigned appropriateness scores were excluded from the analyses [6, 7].

### v. Analysis

#### a. Missing data

In our primary analyses for each quality of life measure, we included all patients with complete data at baseline and 1 year for that particular quality of life indicator. As we anticipated that the same patient may have varying degrees of missingness for each outcome, this would result in potentially different sample sizes for the primary analyses for each outcome. Importantly, to determine if our conclusions were affected by missing data, we created five imputed datasets, using Markov Chain Monte Carlo methods to impute missing data, which were assumed to be missing at random. Regression coefficient estimates were then combined from each imputed dataset to allow for valid statistical inference.

#### b. Model development

Comparison of baseline characteristics between patients in each treatment group was evaluated using the Wilcoxon Rank Sum test for continuous data, and Chi-square or Fisher exact tests for categorical data.

We created six unadjusted linear regression models, each with the scores from the EQ-5D or one of the five domains of the SAQ, treated as a continuous dependent variable. The unit of analysis for all models was the individual patient. To account for the repeated measures per patient, we used generalized estimating equation (GEE) methods, with each patient having both a baseline and a 1-year score. Variables included in the model were time (baseline versus 1 year), treatment strategy (medical therapy versus revascularization), appropriateness category (appropriate versus inappropriate versus uncertain) and all 2-way and 3-way interactions between time, appropriateness category and treatment strategy. In this manner, we were able to contrast quality of life changes from baseline to 1 year for patients treated with revascularization compared to medical therapy alone, within each appropriateness category. Finally, fully adjusted models comparing within and between group changes over time were developed as above, by forcing in all baseline characteristics into the regression. In these models, we adjusted for factors that may impact the quality of life, independent of the choice of revascularization. These included baseline age, gender, LV function, serum creatinine, presence of diabetes mellitus, hypertension, hyperlipidemia, stroke, peripheral vascular disease, smoking, malignancy, heart failure, in addition to having a prior MI, CABG or PCI.

### Sensitivity analyses

Given the higher clinical threshold for considering CABG, as opposed to PCI, as well as the potential for differential benefit between the two treatments, we repeated all the adjusted models, stratified by the type of revascularization modality.

Statistical analyses were performed using SAS version 9.3 (SAS corporation). Results were considered statistically significant with a 2-sided p-value of <0.05.

## Results

During the 1 year of patient accrual, 761 consecutive patients were enrolled. Of these, 282 patients had either mild coronary disease or normal coronaries arteries. A further 54 (7.1%) patients could not be assigned an appropriateness score because they had missing data on a previous stress testing. Our final cohort consisted of 425 patients, with 295 (69.4%) patients treated with revascularization within 90 days of their index angiogram. Of these, 78 (26.4%) patients underwent CABG, with a mean delay of 16.7 days (median 10, interquartile range 0–29 days), while 217 (73.6%) underwent PCI, at a mean delay of 2.5 days (median 0, interquartile range 0–0 days).

Within the final cohort, 272 (64.0%) had appropriate indications for revascularization, while 57 (13.4%) had inappropriate indications, and 96 (22.6%) had uncertain indications respectively. All of the 57 patients who had inappropriate indications had single or double vessel disease without involvement of the LAD and 40.4% were asymptomatic. The majority (51 patients/89.4%) were patients who were receiving minimal or no anti-anginal medications, with low-risk findings on non-invasive testing and were either asymptomatic (n = 17) or minimally symptomatic with CCS 1–2 (n = 34). The remaining 6 inappropriate patients were all asymptomatic, and had either low-risk non-invasive findings and were on maximal anti-anginal medical therapy (n = 4), or intermediate findings with minimal/no medications (n = 2). In the appropriate indication category, 75% of the patients underwent revascularization. In contrast, 53% of the patients with inappropriate indications underwent revascularization. Of the 96 patients with uncertain indications, 62% underwent revascularization.

The baseline characteristics for the cohort are shown on Table 1. Only 19.8% of patients were asymptomatic, with CCS 0 angina. Of the 425 patients in the cohort, 44.2% had single vessel disease, with 31.3% having double vessel disease and 24.6% having either triple vessel or left main artery disease. As expected, there were substantial differences between appropriateness categories in terms of the distribution of symptoms and anatomy, given that these are components of the appropriate use criteria. There were no statistically significant differences in the distribution of LV function. Only 26.1% of the overall cohort were on maximal anti-anginal therapy. The use of statin, β-blockers and ACE inhibitor/ARB medications were high among the cohort, with no differences between appropriateness groups (Table 1). Over a 1- year follow-up period mortality after the index angiogram was 2.6%; 9.9% of the cohort had a repeat MI within 1 year, while 10.4% underwent repeat PCI/CABG within 1 year. There were no differences among the three appropriateness categories in these outcomes; however, patients who were vascularized had a lower unadjusted mortality, but higher rates of both readmission for MI and repeat revascularization.

**Table 1:** **Baseline Characteristics**

Variable	TOTAL N = 425	Appropriate N = 272	Inappropriate N = 57	Uncertain N = 96	P-value	Medical N = 130	Revascularization N = 295	P-value
Age Mean ± SD	65.34 ± 10.86	66.04 ± 11.04	64.11 ± 10.90	64.09 ± 10.23	0.852	65.52 ± 11.69	65.26 ± 10.49	0.823
Male	79.1%	80.1%	78.9%	76.0%	0.123	84.6%	76.6%	0.155
Diabetes	35.1%	38.2%	21.1%	34.4%	0.047	36.9%	34.2%	0.593
Hypertension	74.8%	75.0%	73.7%	75.0%	0.475	80.8%	72.2%	0.152
Hyperlipidemia	89.9%	89.7%	89.5%	90.6%	0.443	90.8%	89.5%	0.764
Smoking	60.0%	62.1%	52.6%	58.3%	0.409	53.1%	3.1%	0.061
Symptom Severity								
CCS 0	19.8%	12.1%	40.4%	29..2%	<0.001	23.1%	18.3%	0.029
CCS 1-2	48.9%	44.1%	59.6%	56.3%		54.6%	46.4%	
CCS 3-4	31.3%	43.8%	0%	14.6%		22.3%	35.3%	
LV ejection Fraction								
> = 60%	56.0%	54.4%	68.4%	53.1%	0.371	48.5%	59.3%	0.039
40-59%	21.9%	22.4%	14.0%	25.0%		30.0%	18.3%	
20-39%	9.4%	11.0%	8.8%	5.2%		10.0%	9.2%	
<=20%	0.7%	0.7%	0.0%	1.0%		1.5%	0.3%	
NA	12.0%	11.4%	8.8%	15.6%		10.0%	12.9%	
Coronary Anatomy								
1 -or 2 vessel CAD without involvement of proximal LAD	55.1%	40.8%	100.0%	68.8%	<.001	63.8%	51.2%	0.016
Chronic total occlusion of 1 major epicaridial coronary artery without other coronary stenoses	0.5%	0.7%	0.0%	0.0%	0.568	0.0%	0.7%	0.347
1- vessel CAD involving the proximal LAD	8.0%	8.8%	0.0%	10.4%	0.051	4.6%	9.5%	0.088
2-vessel CAD involving the proximal LAD	4.7%	3.7%	0.0%	10.4%	0.005	4.6%	4.7%	0.953
3-vessel CAD (no left main)	18.8%	25.7%	0.0%	10.4%	<.001	17.7%	19.3%	0.692
left main stenosis	13.4%	21.0%	0.0%	0.0%	<.001	9.2%	15.3%	0.093
Previous CABG	16.7%	19.9%	1.8%	16.7%	0.005	28.5%	11.5%	<.001
Previous CHF	6.4%	6.6%	3.5%	7.3%	0.74	12.3%	3.7%	0.003
Previous PCI	29.9%	29.4%	29.8%	31.3%	0.159	34.6%	27.8%	0.11
Prior MI	34.8%	33.8%	28.1%	41.7%	0.236	43.1%	31.2%	0.043
PVD	12.5%	11.8%	12.3%	14.6%	0.806	13.8%	11.9%	0.553
Creatinine Mean ± SD	95.92 ± 68.21	90.89 ± 23.96	94.46 ± 44.47	111.04 ± 132.88	0.221	106.38 ± 106.79	91.31 ± 40.45	0.971
Dialysis	1.4%	0.7%	1.8%	3.1%	0.227	2.3%	1.0%	0.299
Stroke	9.6%	9.9%	14.0%	6.3%	0.351	8.5%	10.2%	0.831
Dementia	0.7%	0.7%	0.0%	1.0%	0.341	0.8%	0.7%	0.102
Cancer	10.4%	11.8%	8.8%	7.3%	0.703	7.7%	11.5%	0.01
COPD	7.5%	8.8%	7.0%	4.2%	0.327	(4.6%	8.8%	0.131
Medications								
% on Maximal Anti-anginal Therapy	26.1%	32.0%	7.0%	20.8%	<.001	20.8%	28.5%	0.096
ACE inhibitor/ARB	67.5%	66.9%	68.4%	68.8%	0.936	72.3%	65.4%	0.163
Statin	82.1%	83.1%	82.5%	79.2%	0.688	81.5%	82.4%	0.836
Β-Blocker	66.4%	68.8%	56.1%	65.6%	0.184	71.5%	64.1%	0.133
Treatment Strategy								
CABG	18.4%	21.7%	14.0%	11.5%	0.002	0.0%	26.4%	<.001
Medical	30.6%	24.6%	47.4%	37.5%		130 100.0%	0.0%	
PCI	51.1%	53.7%	38.6%	51.0%		0.0%	73.6%	
Outcomes								
1- year Mortality	2.6%	2.6%	0.0%	4.2%	0.292	5.4%	1.4%	0.016
1-year Readmission for MI	9.9%	10.3%	7.0%	10.4%	0.738	5.4%	11.9%	0.039
1-year repeat PCI/CABG	10.4%	12.5%	3.5%	8.3%	0.098	5.4%	12.5%	0.026

*SD*: standard deviation; *CCS*: Canadian Cardiovascular Society; *LV*: left ventricular; *CABG*: coronary artery bypass grafting; *PCI*: percutaneous coronary intervention; *MI*: myocardial infarction; *PVD*: peripheral vascular disease; *COPD*: chronic obstructive lung disease; *CHF*: Congestive heart failure; *ARB*: angiotensin receptor blocker.

### Quality of life

There was varying degrees of missing data for the six quality of life measures at the two time points of our study. As seen in (Additional file 1: Table S1), only 4.7% (n = 20) of the patients were missing baseline angina frequency scores, but almost 50% were missing angina stability at 1 year. Of the 425 patients, only 113 patients had complete data on all six quality of life measures for both time points. In (Additional file 1: Table S1), the sample size for the primary analyses for each quality indicator is shown. As expected this varied from 252 with complete data on EQ-5D to 213 with complete data on angina stability. Comparison of the patients with missing quality of life measures and those with complete data is found in (Additional file 1: Table S2). The groups were similar with no differences in baseline characteristics. In particular, the proportion of patients that had appropriate, uncertain and inappropriate indications was similar for the complete dataset and that with missing data (Additional file 1: Table S2).

**Figure 1:**
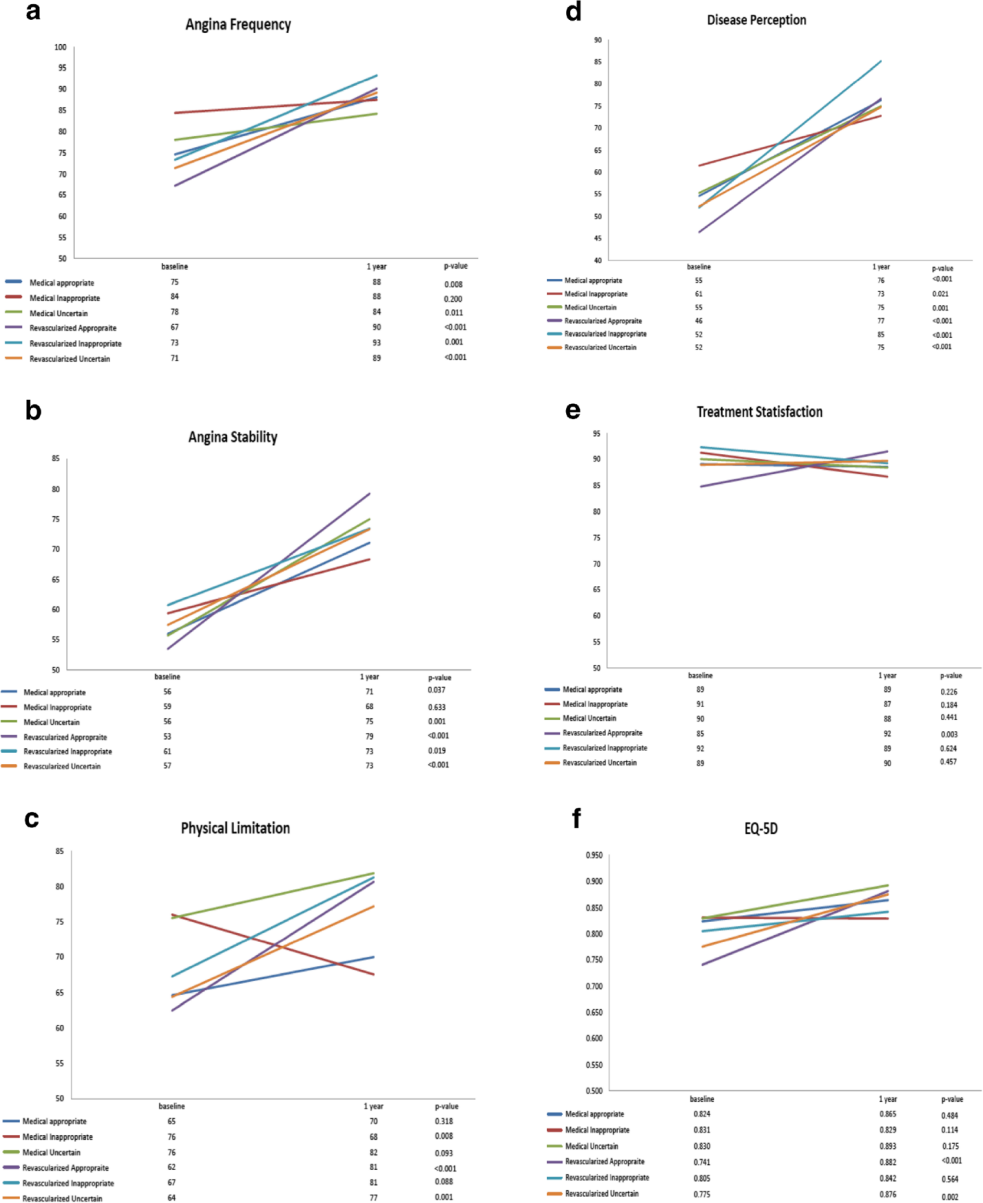
**Changes in quality of life from baseline to 1 year. a**-**f**: Changes in quality of life from baseline to 1 year are shown for patients categorized by treatment strategy and appropriateness category. P-values are based on per patient changes over time. For angina frequency (Figure 1a), higher scores represent less frequent angina.

Summary results for our primary analysis on patients with complete data are shown on Figure 1a-f. As is apparent, most quality of life indices had either statistically significant improvements or remained constant over the 1 year, regardless of appropriateness indication or treatment strategy. Indeed, we did not find a gradient between the appropriate, uncertain or inappropriate criteria. The exception was in patients treated medically who had inappropriate indications for revascularization; these patients had a significant worsening in their mean physical limitation scores over the 1 year (Figure 1c). Angina frequency and angina stability improved significantly for all patients, except those who had inappropriate indications and were treated medically (Figure 1a and 1b respectively). Disease perception improved significantly over the course of the 1-year follow-up in all groups (Figure 1d). Treatment satisfaction only improved significantly for patients with appropriate indications who were revascularized. In (Additional file 1: Table S3), we compared the proportion of patients who had substantial improvements (>20 point) over the 1-year of follow-up in the SAQ sub-scales. Patients who were revascularized had a higher proportion of patients with substantial improvements in angina frequency (33.9% vs 15.4%; p < 0.001), physical limitation (20% vs 5.4%; p < 0.001) and disease perception 31.9% vs 20% p = 0.012). However, across the appropriate use criteria, the only difference was in physical limitation (18.0% for appropriate vs 5.3% inappropriate p = 0.052).

A comparison of baseline quality of life measures for the patients with complete and missing data, stratified by appropriateness category is found in (Additional file 1: Table S4). There were no significant differences in baseline scores for the five angina specific measures from the SAQ, except for physical limitation where patients with missing values had lower scores. There were also differences in generic quality of life as measured by the EQ-5D. Patients with missing data tended to have lower baseline quality of life as measured in the EQ-5D.

#### Appropriate indications

In the appropriate category, patients who were treated with revascularization tended to have lower baseline quality of life scores compared to medical therapy patients (Figure 1a-f). However, when comparing risk-adjusted changes over time (Table 2), there were statistically significant differences in the angina stability, physical limitation, disease perception and treatment satisfaction SAQ sub-categories, as well as in generic EQ-5D in patients with appropriate indications for revascularization who were revascularized compared to those who were treated medically. In each of these categories, patients who had appropriate indications and were revascularized had incrementally greater improvements in quality of life compared to those who were treated medically.

**Table 2:** **Comparisons of Changes in Quality of Life Indices from baseline to 1 year**

Comparison		Unadjusted (95% CI)	p-value	Adjusted (95% CI)	p-value
ANGINA FREQUENCY: n = 247					
*Indication*	*Therapy*				
Appropriate	Revascularization VS medical therapy	10.2 (-0.6,20.9)	0.063	9.8 (-1.6,21.2)	0.093
Inappropriate	Revascularization VS medical therapy	13.6 (-2.1,29.3)	0.090	10.0 (-6.2,26.2)	0.227
Uncertain	Revascularization VS medical therapy	10.6 (-1.7,22.9)	0.09	11.3 (-1.3,24.0)	0.080
ANGINA STABILITY: n = 213					
*Indication*	*Therapy*				
Appropriate	Revascularization VS medical therapy	14.6 (0.8,28.3)	0.038	14.6 (0.8,28.3)	0.038
Inappropriate	Revascularization VS medical therapy	13.0 (-17.3,43.3)	0.401	9.7 (-20.4,39.7)	0.529
Uncertain	Revascularization VS medical therapy	-9.4 (-35.1,16.3)	0.475	-7.7 (-34.4,19.0)	0.571
PHYSICAL LIMITATION: n = 239					
*Indication*	*Therapy*				
Appropriate	Revascularization VS medical therapy	10.1 (1.8,18.5)	0.017	9.3 (0.7,17.8)	0.034
Inappropriate	Revascularization VS medical therapy	18.3 (5.8,30.8)	0.004	17.7 (3.9,31.6)	0.012
Uncertain	Revascularization VS medical therapy	19.3 (8.6,29.9)	<0.001	19.2 (8.2,30.1)	<0.001
DISEASE PERCEPTION: n = 243					
*Indication*	*Therapy*				
Appropriate	Revascularization VS medical therapy	14.4 (5.6,23.1)	0.001	12.7 (4.3,21.1)	0.003
Inappropriate	Revascularization VS medical therapy	26.0 (13.6,38.5)	<0.001	26.9 (13.6,40.3)	<0.001
Uncertain	Revascularization VS medical therapy	7.2 (-4.8,19.1)	0.239	6.5 (-5.7,18.8)	0.294
TREATMENT SATISFACTION: n = 246					
*Indication*	*Therapy*				
Appropriate	Revascularization VS medical therapy	7.6 (2.1,13.1)	0.007	7.6 (1.9,13.2)	0.009
Inappropriate	Revascularization VS medical therapy	4.5 (-4.1,13.1)	0.302	6.0 (-2.5,14.5)	0.167
Uncertain	Revascularization VS medical therapy	1.2 (-8.4,10.9)	0.802	1.7 (-8.1,11.4)	0.733
EQ_5D: n = 252					
*Indication*	*Therapy*				
Appropriate	Revascularization VS medical therapy	0.095 (0.024,0.165)	0.008	0.073 (0.003,0.144)	0.040
Inappropriate	Revascularization VS medical therapy	-0.010 (-0.0168,0.148)	0.902	-0.009 (-0.189,0.171)	0.920
Uncertain	Revascularization VS medical therapy	0.062 (-0.012,0.135)	0.099	0.063 (-0.014,0.139)	0.107

#### Inappropriate and uncertain indications

Interestingly, patients who had inappropriate indications for revascularization also had greater improvements in disease perception and physical limitation when treated with revascularization compared to medical therapy (Table 2). Similarly, patients with uncertain indications had greater improvements in physical limitation with revascularization.

### Sensitivity analyses

Multiple imputation was used to account for missing data. As seen in the (Additional file 1: Table S5), although there were no qualitative differences in the trend of our results when compared to our primary analysis, the findings were no longer statistically significant.

The risk-adjusted models, stratified by revascularization modality are found in (Additional file 1: Table S6). We found similar findings for patients treated with CABG, as compared to the overall primary results for both patients with appropriate and inappropriate indications. However, for patients treated with PCI who had appropriate indications, improvements in quality of life were limited to treatment satisfaction and disease perception.

## Discussion

We found that the majority of patients with significant SIHD who underwent coronary angiography had appropriate indications for revascularization. Approximately three quarters of these patients underwent subsequent revascularization. Most patients had improvements in their self-reported quality of life in the first year after coronary angiography. Although patients with appropriate indications for revascularization derived greater improvements with revascularization compared to medical therapy, improvements were also seen for revascularized patients with uncertain and inappropriate indications, albeit in fewer quality of life sub-categories. However, these findings were not robust in sensitivity analyses that accounted for missing data with multiple imputations, and were most apparent in patients who underwent CABG, as opposed to those who were revascularized with PCI. Although appropriate use criteria appear to identify appropriate candidates for revascularization, our study raises doubts as to the utility of these criteria in excluding patients who are not candidates for revascularization.

There has been substantial interest in the development for appropriate use criteria for diagnostic tests and therapies, particularly in cardiovascular disease [11–13]. These appropriate use criteria are developed through the expert consensus, and as such it is important to evaluate if the criteria translate into improved outcomes. Previous work from our group has validated the appropriateness criteria for revascularization by demonstrating that in SIHD patients who have appropriate indications, medical therapy was associated with worse outcomes when compared to revascularization, with a 39% increased hazard of death or repeat myocardial infarction over 3 years of follow-up [14]. Moreover, we found that revascularization was underutilized, with about 30% of patients with appropriate indications not receiving revascularization [14]. However, we did not find worse outcomes in revascularized patients who had uncertain or inappropriate indications, a finding consistent with other studies [14, 15]. We extend this previous work in the current study by evaluating the impact of under and overutilization of coronary revascularization on quality of life.

Quality of life is increasingly used as a patient-centered endpoint in cardiovascular disease studies, especially in SIHD, where invasive therapy is indicated for the relief of symptoms [16, 17]. In the landmark Clinical Outcomes Utilizing Revascularization and Aggressive Drug Evaluation (COURAGE) trial, both patients treated with optimal medical therapy and those revascularized with PCI, had marked improvements in their quality of life as measured on the SAQ [18]. Patients who were treated with PCI had small incremental benefits over medical therapy, which was most prominent in the patient subgroup with more severe angina symptoms at baseline [18]. This is consistent with other evidence that has shown that the most important predictor of quality of life improvements post PCI is the severity of baseline angina [19]. Our study adds to these previous findings. Similar to the COURAGE trial, we found that the majority of patients tended to have improvements in quality of life. The greater benefit associated with revascularization compared to medical therapy seen in our study, in contrast to the COURAGE trial, maybe related to the relatively low proportion of patients in our cohort who on maximum anti-anginal therapy.

In patients with appropriate indications, we have reinforced the validation of the appropriate category, given the improved quality of life we observed for patients treated with revascularization who had appropriate indications. In combination with our previous work showing improved clinical outcomes for these patients with revascularization, this supports the use of the appropriate use criteria in identifying suitable candidates for revascularization.

The interpretation of the quality of life changes in the uncertain and inappropriate categories is complex. The rationale underlying the inappropriate indication category is that revascularization in these patients should not be of any benefit at all, and potentially harmful because these patients have minimal chest pain and good baseline function. However, despite the relatively small number of patients in these categories, we found that revascularization in patients with both uncertain and inappropriate criteria appeared to have greater improvements in quality of life in some of the SAQ sub-categories when compared to medical therapy. This suggests the appropriate use criteria may need greater refinement in order to better exclude patients who are indeed poor candidates for revascularization. The lack of discrimination for the appropriateness guidelines for clinical benefit may relate to the inclusion of symptomatic patients in the inappropriate category. Indeed, of the 57 patients with inappropriate indications, 60% had symptoms. Potential refinements may be the inclusion of quality of life as measured on instruments such as the SAQ, as opposed to CCS class, in order to better identify patients with minimal symptoms who would not benefit from revascularization.

Our study must be interpreted in the context of several limitations that merit discussion. First, despite our best efforts, a considerable portion of enrolled patients did not complete all the quality of life surveys. We evaluated the impact of missing data in the sensitivity analyses through multiple imputations. Although it is reassuring that the qualitative direction of our results were maintained, the comparisons between treatment strategies within appropriateness categories were not longer statistically significant. Second, our analysis was restricted to patients who survived at least a year after their index coronary angiogram. This limitation is mitigated by the fact that we were evaluating a SIHD population and therefore, we would not expect a high mortality rate. In addition, it is notable that only approximately a quarter of our patients were on optimal medical therapy, reflecting the inadequacy of routine medical care in real world practice. Our cohort consisted of patients recruited at a single, large tertiary academic hospital in Ontario, Canada. Indeed, previous work from our group suggests that there maybe a higher threshold for invasive testing in Canada, and as such our results may not be generalizable to other jurisdictions or patient populations [4]. We were limited in the anatomical data available to the location of the lesions. Although this was sufficient to assign patients to appropriateness categories, we were not able to account for the complexity of anatomy, such as the presence of calcification or bifurcation status which may have impacted the decision to revascularize. Finally, this is an observational study designed to evaluate associations between treatment strategy, appropriate use criteria and outcomes. We cannot establish causality, and so our work should be considered hypothesis generating and not conclusive. We believe that our results reinforce the need for an adequately powered randomized trial to evaluate the benefit of determining treatment strategy based on appropriate use criteria.

## Conclusions

In conclusion, we found that coronary revascularization is associated with greater improvements in quality of life compared to medical therapy, in particular in patients with appropriate indications. However, refinements are needed in the appropriate use criteria to better exclude patients who would not derive benefit from revascularization.

## Electronic supplementary material

Additional file 1: **Appendix.** (DOCX 44 KB)
