# Supplementary material for: Association between appropriateness of coronary revascularization and quality of life in patients with stable ischemic heart disease
Source: BMC Cardiovasc Disord. 2014 Oct 4;14:137. doi: 10.1186/1471-2261-14-137 (PMC4195906; doi:10.1186/1471-2261-14-137)
Supplement: Supplementary file 1 — Additional file 1: Appendix. (DOCX 44 KB) [file 12872_2014_784_MOESM1_ESM.docx]

Appendix:

Table 1: Degree of Missingness by Quality of Life Measure

|  | Sample Size for Primary Analyses | Number (%) of missing | |
| --- | --- | --- | --- |
|  |  | baseline | 1 year |
| Angina Frequency | 247 | 20 (4.7%) | 178 (41.9%) |
| Angina Stability | 213 | 142 (33.4%) | 212 (49.9%) |
| Physical Limitation | 239 | 23 (5.4%) | 186 (43.8%) |
| Disease Perception | 243 | 48 (11.3%) | 182 (42.8%) |
| Treatment Satisfaction | 246 | 32 (7.5%) | 179 (42.1%) |
| EQ-5D | 252 | 57 (13.4%) | 173 ((40.7%) |

Table 2: Baseline Characteristics of Patients with Missing Data for Quality of Life

|  |  | Non-missing QOL | Missing Any QOL | TOTAL | P-VALUE |
| --- | --- | --- | --- | --- | --- |
|  |  | N=113 | N=312 | N=425 |  |
|  |  |  |  |  |  |
| Appropriateness score category | APPROPRIATE | 71 (62.8%) | 201 (64.4%) | 272 (64.0%) | 0.643 |
|  | INAPPROPRIATE | 18 (15.9%) | 39 (12.5%) | 57 (13.4%) |  |
|  | UNCERTAIN | 24 (21.2%) | 72 (23.1%) | 96 (22.6%) |  |
|  |  |  |  |  |  |
| Treatment Strategy | Medical | 34 (30.1%) | 96 (30.8%) | 130 (30.6%) | 0.893 |
|  | Revascularization | 79 (69.9%) | 216 (69.2%) | 295 (69.4%) |  |
|  |  |  |  |  |  |
| Treatment: CABG, PCI, Med | CABG | 17 (15.0%) | 61 (19.6%) | 78 (18.4%) | 0.505 |
|  | Medical | 34 (30.1%) | 96 (30.8%) | 130 (30.6%) |  |
|  | PCI | 62 (54.9%) | 155 (49.7%) | 217 (51.1%) |  |
|  |  |  |  |  |  |
| Age | Mean ± SD | 65.33 ± 11.17 | 65.34 ± 10.76 | 65.34 ± 10.86 | 0.991 |
| Gender | Male | 92 (81.4%) | 244 (78.2%) | 336 (79.1%) | 0.17 |
| Diabetes Mellitus |  | 37 (32.7%) | 112 (35.9%) | 149 (35.1%) | 0.547 |
| Hypertension | YES | 86 (76.1%) | 232 (74.4%) | 318 (74.8%) | 0.793 |
| hyperlipidemia | YES | 106 (93.8%) | 276 (88.5%) | 382 (89.9%) | 0.254 |
| smoking | YES | 68 (60.2%) | 187 (59.9%) | 255 (60.0%) | 0.577 |
| CCS Class | ccs class 0 | 18 (15.9%) | 66 (21.2%) | 84 (19.8%) | 0.365 |
|  | ccs class:1,2 | 61 (54.0%) | 147 (47.1%) | 208 (48.9%) |  |
|  | ccs class:3,4 | 34 (30.1%) | 99 (31.7%) | 133 (31.3%) |  |
|  |  |  |  |  |  |
| Left Ventricular Ejection | >=60% | 66 (58.4%) | 172 (55.1%) | 238 (56.0%) | 0.698 |
|  | 40-59% | 20 (17.7%) | 73 (23.4%) | 93 (21.9%) |  |
|  | 20-39% | 13 (11.5%) | 27 (8.7%) | 40 (9.4%) |  |
|  | <=20% | 1 (0.9%) | 2 (0.6%) | 3 (0.7%) |  |
| Previous CABG | YES | 21 (18.6%) | 50 (16.0%) | 71 (16.7%) | 0.581 |
| Previous CHF | YES | 7 (6.2%) | 20 (6.4%) | 27 (6.4%) | 0.752 |
| Previous PCI | YES | 36 (31.9%) | 91 (29.2%) | 127 (29.9%) | 0.730 |
| Prior MI | YES | 37 (32.7%) | 111 (35.6%) | 148 (34.8%) | 0.664 |
| PVD | YES | 19 (16.8%) | 34 (10.9%) | 53 (12.5%) | 0.19 |
| Creatinine | Mean ± SD | 95.52 ± 58.61 | 96.06 ± 71.46 | 95.92 ± 68.21 | 0.942 |
| Dialysis |  | 0 (0.0%) | 6 (1.9%) | 6 (1.4%) | 0.138 |
| Stroke | YES | 12 (10.6%) | 29 (9.3%) | 41 (9.6%) | 0.918 |
| Dementia | YES | 0 (0.0%) | 3 (1.0%) | 3 (0.7%) | 0.4 |
| Cancer | YES | 11 (9.7%) | 33 (10.6%) | 44 (10.4%) | 0.826 |
| COPD |  | 9 (8.0%) | 23 (7.4%) | 32 (7.5%) | 0.838 |
| % on Maximal Anti-anginal Therapy |  | 28 (24.8%) | 83 (26.6%) | 111 (26.1%) | 0.705 |
| ACE inhibitor/ARB |  | 78 (69.0%) | 209 (67.0%) | 287 (67.5%) | 0.692 |
| Statin |  | 96 (85.0%) | 253 (81.1%) | 349 (82.1%) | 0.358 |
| Β-Blocker |  | 75 (66.4%) | 207 (66.3%) | 282 (66.4%) | 0.996 |
| Died within 1 year after index |  | 0 (0.0%) | 11 (3.5%) | 11 (2.6%) | 0.043 |
| Re-admission MI within 1 year |  | 12 (10.6%) | 30 (9.6%) | 42 (9.9%) | 0.759 |
| Re-admission PCI/CABG within 1 year |  | 11 (9.7%) | 33 (10.6%) | 44 (10.4%) | 0.801 |

SD: standard deviation; CCS: Canadian Cardiovascular Society; LV: left ventricular; CABG: coronary artery bypass grafting; PCI: percutaneous coronary intervention; MI: myocardial infarction; PVD: peripheral vascular disease; COPD: chronic obstructive lung disease; CHF: Congestive heart failure; ARB: angiotensin receptor blocker

Table 3: Proportion of Patients with Substantial Improvements in Quality of Life Scores

|  | TOTAL | APPROPRIATE | INAPPROPRIATE | UNCERTAIN | P-VALUE | Medical | Revascularization | P-VALUE |
| --- | --- | --- | --- | --- | --- | --- | --- | --- |
|  | N=425 | N=272 | N=57 | N=96 |  | N=130 | N=295 |  |
| **Substantial Improvement >20** | | | | | | | | |
| Angina Frequency | 120 (28.2%) | 80 (29.4%) | 15 (26.3%) | 25 (26.0%) | 0.772 | 20 (15.4%) | 100 (33.9%) | <.001 |
| Angina Stability | 85 (20.0%) | 61 (22.4%) | 8 (14.0%) | 16 (16.7%) | 0.23 | 20 (15.4%) | 65 (22.0%) | 0.114 |
| Physical Limitation | 66 (15.5%) | 49 (18.0%) | 3 (5.3%) | 14 (14.6%) | 0.052 | 7 (5.4%) | 59 (20.0%) | <.001 |
| Disease Perception | 120 (28.2%) | 81 (29.8%) | 16 (28.1%) | 23 (24.0%) | 0.552 | 26 (20.0%) | 94 (31.9%) | 0.012 |
| Treatment Satisfaction | 15 (3.5%) | 13 (4.8%) | 0 (0.0%) | 2 (2.1%) | 0.141 | 2 (1.5%) | 13 (4.4%) | 0.14 |

Table 4: Baseline Quality of Life Scores in Patients with Missing vs. Non-Missing Data

| **Overall** | | | | |
| --- | --- | --- | --- | --- |
|  | **Non-missing QOL** | **Missing Any QOL** | **TOTAL** | **P-VALUE** |
|  | **N=113** | **N=312** | **N=425** |  |
| Angina Frequency | 71.95 ( 25.21) | 71.16 (25.82) | 71.38 (25.62) | 0.783 |
| Angina Stability | 52.88 (24.72) | 57.06 (24.21) | 55.39 (24.46) | 0.159 |
| Physical Limitation | 68.18 (21.87) | 64.27 (25.39) | 65.37 (24.49) | 0.150 |
| Disease Perception | 54.42 (22.43) | 48.94 (23.98) | 50.59 (23.63) | 0.039 |
| Treatment Satisfaction | 88.68 (13.86) | 86.84 (15.89) | 87.37 (15.34) | 0.282 |
| EQ-5D | 0.80 (0.12) | 0.76 (0.17) | 0.78 ( 0.16) | 0.041 |
| **Appropriate indication** | | | | |
| **VARIABLE** | **Non-missing QOL** | **Missing Any QOL** | **TOTAL** | **P-VALUE** |
|  | **N=71** | **N=201** | **N=272** |  |
| Angina Frequency | 71.69 (25.24) | 67.98 ( 26.04) | 69.00 (25.83) | 0.303 |
| Angina Stability | 50.70 ( 21.54) | 56.03 (23.92) | 54.01(23.13) | 0.126 |
| Physical Limitation | 65.43 (20.79) | 62.04 (25.02) | 62.98 (23.93) | 0.311 |
| Disease Perception | 52.52 ( 22.40) | 46.70 (24.26) | 48.38 ( 23.83) | 0.082 |
| Treatment Satisfaction | 87.12 (15.23) | 85.30 (16.77) | 85.81 (16.35) | 0.428 |
| EQ-5D | 0.80 (0.12) | 0.75(0.18) | 0.76 (0.17) | 0.026 |
| **Inappropriate indication** | | | | |
| **VARIABLE** | **Non-missing QOL** | **Missing Any QOL** | **TOTAL** | **P-VALUE** |
|  | **N=18** | **N=39** | **N=57** |  |
| Angina Frequency | 77.22 (23.96) | 79.41 (22.69) | 78.65 (22.93) | 0.747 |
| Angina Stability | 61.11 (29.98) | 59.21 ( 25.29) | 60.14 (27.30) | 0.836 |
| Physical Limitation | 78.32 (18.21) | 68.33 (24.69) | 71.72 (23.02) | 0.136 |
| Disease Perception | 64.12 (16.86) | 52.28 (22.23) | 56.63 (21.04) | 0.057 |
| Treatment Satisfaction | 94.91 ( 8.46) | 90.09 ( 13.56) | 91.79 (12.14) | 0.178 |
| EQ-5D | 0.83 (0.07) | 0.81 (0.15) | 0.82 (0.12) | 0.524 |
| **Uncertain indication** | | | | |
| **VARIABLE** | **Non-missing QOL** | **Missing Any QOL** | **TOTAL** | **P-VALUE** |
|  | **N=24** | **N=72** | **N=96** |  |
| Angina Frequency | 68.75 (26.43) | 75.71 (25.40) | 73.94 (25.70) | 0.254 |
| Angina Stability | 53.13 (28.85) | 59.29 (25.06) | 56.78 (26.60) | 0.387 |
| Physical Limitation | 68.69 (25.77) | 68.26 (26.36) | 68.37 (26.07) | 0.945 |
| Disease Perception | 52.78 (25.02) | 53.81 (23.51) | 53.51 (23.81) | 0.859 |
| Treatment Satisfaction | 88.63 (11.78) | 89.62 (13.81) | 89.34 (13.22) | 0.757 |
| EQ-5D | 0.79 (0.17) | 0.80 ( 0.16) | 0.80 (0.16) | 0.804 |

Table 5: Multiple Imputations Analyses

| Comparison | | **ESTIMATE** | (95% CI) | p-value |
| --- | --- | --- | --- | --- |
| ANGINA FREQUENCY | | | | |
| *Indication* | *Therapy* |  |  |  |
| Appropriate | Revascularization VS medical therapy | 7.2 | (-3.2,17.6) | 0.169 |
| Inappropriate | Revascularization VS medical therapy | 9.6 | (-4.3,23.5) | 0.174 |
| Uncertain | Revascularization VS medical therapy | 6.6 | (-6.3,19.5) | 0.311 |
| ANGINA STABILITY | | | | |
| *Indication* | *Therapy* |  |  |  |
| Appropriate | Revascularization VS medical therapy | 9.6 | (-0.9,20.1) | 0.073 |
| Inappropriate | Revascularization VS medical therapy | 4.5 | (-16.2,25.2) | 0.664 |
| Uncertain | Revascularization VS medical therapy | 6.4 | (-8.5,21.3) | 0.397 |
| PHYSICAL LIMITATION | | | | |
| *Indication* | *Therapy* |  |  |  |
| Appropriate | Revascularization VS medical therapy | 6.4 | (-2.0,14.8) | 0.133 |
| Inappropriate | Revascularization VS medical therapy | 13.7 | (0.32,27.1) | 0.045 |
| Uncertain | Revascularization VS medical therapy | 10.3 | (-1.0,21.7) | 0.074 |
| DISEASE PERCEPTION | | | | |
| *Indication* | *Therapy* |  |  |  |
| Appropriate | Revascularization VS medical therapy | 7.6 | (-0.1,15.3) | 0.054 |
| Inappropriate | Revascularization VS medical therapy | 8.9 | (-9.1,26.9) | 0.318 |
| Uncertain | Revascularization VS medical therapy | 5.3 | (-6.2,16.7) | 0.363 |
| TREATMENT SATISFACTION | | | | |
| *Indication* | *Therapy* |  |  |  |
| Appropriate | Revascularization VS medical therapy | 5.3 | (-0.4,11.0) | 0.067 |
| Inappropriate | Revascularization VS medical therapy | 0.3 | (-10.2,10.8) | 0.953 |
| Uncertain | Revascularization VS medical therapy | 3.9 | (-5.7,13.6) | 0.408 |
| EQ_5D | | | | |
| *Indication* | *Therapy* |  |  |  |
| Appropriate | Revascularization VS medical therapy | 0.058 | (-0.003,0.120) | 0.061 |
| Inappropriate | Revascularization VS medical therapy | -0.004 | (-0.124,0.117) | 0.953 |
| Uncertain | Revascularization VS medical therapy | 0.038 | (-0.029,0.106) | 0.263 |

Table 6: Quality of Life Changes in PCI vs. CABG Patients

| **Comparison** | | | **PCI vs Medical**  **(95% CI)** | **p-value** | **CABG vs Medical**  **(95% CI)** | **p-value** |
| --- | --- | --- | --- | --- | --- | --- |
|  | ANGINA FREQUENCY | | | | | |
| *Indication* | | *Therapy* |  |  |  |  |
| Appropriate | | Revascularization VS medical therapy | 9.7  (-2.3,21.7) | 0.116 | 9.9  (-2.7,22.6) | 0.123 |
| Inappropriate | | Revascularization VS medical therapy | 11.1  (-5.1,27.2) | 0.180 | 8.6  (-18.1,35.2) | 0.528 |
| Uncertain | | Revascularization VS medical therapy | 13.0  (0.1,25.9) | 0.047 | -1.1  (-31.7,29.5) | 0.943 |
|  | ANGINA STABILITY | | | | | |
| *Indication* | | *Therapy* |  |  |  |  |
| Appropriate | | Revascularization VS medical therapy | 11.6  (-2.5,25.8) | 0.109 | 25.0  (5.9,44.0) | 0.010 |
| Inappropriate | | Revascularization VS medical therapy | 11.6  (-22.8,45.1) | 0.496 | 6.3  (-27.0,39.5) | 0.713 |
| Uncertain | | Revascularization VS medical therapy | -7.6  (-35.0,19.8) | 0.587 | -9.4  (-30.5,11.7) | 0.384 |
|  | PHYSICAL LIMITATION | | | | | |
| *Indication* | | *Therapy* |  |  |  |  |
| Appropriate | | Revascularization VS medical therapy | 7.9  (-1.1,16.8) | 0.086 | 13.1  (2.4,23.7) | 0.016 |
| Inappropriate | | Revascularization VS medical therapy | 11.6  (-3.7,26.9) | 0.138 | 30.0  (10.2,49.8 | <0.001 |
| Uncertain | | Revascularization VS medical therapy | 22.2  (11.5,33.0) | <0.001 | -12.7  (-41.8,16.3) | 0.391 |
|  | DISEASE PERCEPTION | | | | | |
| *Indication* | | *Therapy* |  |  |  |  |
| Appropriate | | Revascularization VS medical therapy | 12.8  (4.0,21.7) | 0.004 | 12.2  (0.0,24.5) | 0.049 |
| Inappropriate | | Revascularization VS medical therapy | 23.3  (5.4,41.2) | 0.010 | 32.7  (19.8,45.5) | <0.001 |
| Uncertain | | Revascularization VS medical therapy | 8.5  (-3.3,20.4) | 0.158 | -46.9  (-55.5,-38.4) | <0.001 |
|  | TREATMENT SATISFACTION | | | | | |
| *Indication* | | *Therapy* |  |  |  |  |
| Appropriate | | Revascularization VS medical therapy | 6.4  (0.6,12.2) | 0.031 | 11.2  (2.320.1) | 0.014 |
| Inappropriate | | Revascularization VS medical therapy | 7.2  (-2.0,16.4) | 0.126 | 4.1  (-4.1,12.3) | 0.327 |
| Uncertain | | Revascularization VS medical therapy | 0.9  (-9.1,10.8) | 0.862 | 9.4  (-3.4,22.3) | 0.151 |
|  | EQ_5D | | | | | |
| *Indication* | | *Therapy* |  |  |  |  |
| Appropriate | | Revascularization VS medical therapy | 0.067  (-0.005,0.139) | 0.069 | 0.097  (0.010,0.183) | 0.028 |
| Inappropriate | | Revascularization VS medical therapy | -0.119  (-0.394,0.156) | 0.397 | 0.10  (-0.07,0.274) | 0.258 |
| Uncertain | | Revascularization VS medical therapy | 0.069  (-0.010,0.148) | 0.088 | -0.029  (-0.071,0.013) | 0.176 |
